# MobileLAMP: A low-cost, portable incubation device for isothermal nucleic acid amplification

**DOI:** 10.1371/journal.pone.0346874

**Published:** 2026-04-16

**Authors:** Mohini Bhupathi, Smitha Hegde, Jennifer C. Molloy, Ganga Chinna Rao Devarapu

**Affiliations:** 1 Rossa Lodge, Rossa Avenue, Bishopstown, Cork, Ireland; 2 Department of Chemical Engineering and Biotechnology, University of Cambridge, United Kingdom; 3 Centre for Advanced Photonics and Process Analysis, Munster Technological University, Cork, Ireland; 4 Tyndall National Institute, Cork, Ireland; Dana-Farber Cancer Institute, UNITED STATES OF AMERICA

## Abstract

Isothermal amplification-based methods for pathogen DNA or RNA detection offer high sensitivity, rapid detection, and the potential for deployment in remote fields and home testing. Consequently, they are emerging as alternatives to PCR and saw a surge in research activity and deployment for the rapid detection of SARS-CoV-2 during the Covid-19 pandemic. The most common isothermal DNA detection methods rely on minimal reagents for DNA amplification and simple hardware that can maintain isothermal conditions and read-out a fluorescent or colorimetric signal. Many researchers globally are working on improving these components based on diverse end-user needs. In this work, we present MobileLAMP, an open-source, 3D-printed incubation device designed for loop-mediated isothermal amplification (LAMP). Composed of off-the-shelf components, MobileLAMP is easily manufacturable and can be powered via any 5V USB source. The device maintains high thermal stability (standard deviation = 0.2°C) across a functional range typically used for LAMP (55–65°C) while consuming only 365 mA of current. Efficacy was demonstrated through the colorimetric detection of SARS-CoV-2 and *Salmonella enterica* serovar Typhi within a 60-minute incubation period. With a total component cost of less than $5, MobileLAMP provides a highly accessible platform for decentralised molecular diagnostics, supporting both distributed manufacturing and field-based applications.

## 1 Introduction

Isothermal amplification has emerged as an alternative to PCR for nucleic acid-based diagnostics over the last two decades. Since then, loop-mediated isothermal amplification (LAMP) [1], recombinase polymerase amplification (RPA) [2], nucleic acid sequence-based amplification (NASBA) [3], strand displacement amplification (SDA), helicase dependent amplification (HDA), etc., have been subject to significant innovation and increasing popularity. As of 2019, LAMP is the most published isothermal method based on Web of Science data [4]. Its simple principle, high sensitivity, rapid results for diagnostic applications, dependence on a single DNA polymerase enzyme with minimal assay components has made it a popular choice for nucleic acid Amplification Testing (NAT) despite drawbacks due to the complexity of primer design, propensity for non-specific amplification and high risk of amplicon contamination.

Isothermal NAT such as LAMP also allow the use of simple and low-cost devices that maintain isothermal conditions, without the need for temperature cycling. Amplification has most often been monitored by fluorescence detection via DNA intercalating dyes and fluorophore-containing probes. LAMP detection methods have extended to visual readouts; without the need for any machines or lateral-flow devices [5]. These simplifications offer promising applications for home-testing, field-testing or use in resource-constrained point-of-care testing. As a result of this flexibility, several LAMP assays have been commercialised for COVID-19 [6] and based on these successes several other LAMP assays are being developed for malaria (US-LAMP) [7], HIV [8] and tuberculosis (TB-LAMP) [9]. One limitation for the proliferation of LAMP outside of medical laboratories is the requirement for an incubation device that maintains a constant temperature. Therefore, several efforts have focused on developing a simple incubator [10–29]. Many of these existing isothermal incubation solutions present trade-offs in terms of cost, complexity, power requirements, or scalability, hindering widespread adoption in resource-limited settings (see Table 1).

**Table 1:** Comparison of MobileLAMP with existing LAMP incubation devices across cost, temperature stability, open-source status, power requirements, and fabrication complexity. Power consumption specifications include wattage (W), voltage (V), and current (A). Fabrication complexity is assessed across five dimensions: 3D printing requirement, CNC machining, custom electronics design, soldering, and programming. Cost values marked with asterisk (*) indicate either partial data or estimated retail prices. NR, not reported in original publication; N/A, not applicable. References are provided in brackets for each device.

Incubation device	Device Cost ($)	Temperature Stability (SD or ±) in °C	Open source	Power Source and Consumption				Fabrication Complexity				
				Electricity Type	Wattage (W)	Voltage (V)	Current (A)	3D printing	CNC	Custom Electronics	Soldering	Programming
**Open-Source Electronic Incubators**												
**MobileLAMP [This Work]**	5	0.2	Yes	USB	1.8	5	0.365	Yes	No	No	No	No
**LAMP Shield [10]**	20	1.4	Yes	Battery	2	7	0.5	No	No	Yes	Yes	Yes
**Non-Instrumented Systems**												
**NINA-LAMP [**11,12**]**	10	2	No	N/A	N/A	N/A	N/A	No	Yes	No	No	No
**T-Cup [13]**	1	3	Yes	N/A	N/A	N/A	N/A	Yes	No	No	No	No
**Repurposed Lab/ Household Devices**												
**Water Circulator [14]**	250*	NR	Yes	Mains AC	NR	120	NR	Yes	No	No	No	No
**Sous-Vide** **[**15–17**]**	65*	0.5	Yes	Mains AC	NR	120	N/A	No	No	No	No	No
**Hot plate [18]**	NR	NR	No	Mains AC	NR	NR	NR	No	No	No	No	No
**Incubator [19]**	NR	NR	No	Mains AC	NR	NR	NR	No	No	No	No	No
**Advanced/ Integrated Portable Platforms**												
**Portable Reader [20]**	NR	NR	No	NR	NR	NR	NR	Yes	No	Yes	Yes	Yes
**qcLAMP [21]**	NR	NR	No	USB	NR	NR	NR	Yes	No	Yes	Yes	Yes
**FABL-8 [22]**	380	0.05	Yes	Mains AC/DC	120	19	6.3	Yes	Yes	Yes	Yes	Yes
**LARI [23]**	1500	0.4	Partial	Mains AC/DC	NR	120	NR	Yes	Yes	Yes	Yes	Yes
**FluoroPLUM [24]**	NR	NR	No	Battery	120	12	10	NR	NR	NR	NR	NR
**MINI [25]**	4900	1	Partial	Mains AC/DC	72	12	6	Yes	Yes	Yes	Yes	Yes
**LAMP-on-a-Chip [26]**	180	0.1	Yes	Mains AC/DC	60	12	5	Yes	Yes	Yes	Yes	Yes
**Colorimetric Lamp [27]**	32	0.65	No	Mains AC/DC	NR	12	NR	Yes	No	Yes	Yes	Yes
**qByte [28]**	60	NR	Yes	USB-C	30	9/12	2.5	Yes	No	Yes	Yes	Yes
**Flash [29]**	12*	NR	Partial	NR	NR	NR	NR	Yes	No	No	NR	No

For instance, some low-cost incubators are entirely passive, relying on exothermic chemical reactions (e.g., NINA-LAMP [11,12], T-Cup [13]), which lack active temperature control, making it difficult to obtain consistent and reliable results. Other electronic DIY solutions such as LAMP Shield [10], while more actively controlled, can still require specialized assembly skills like programming and soldering. Even for repurposed off-the-shelf commercial heating equipment (e.g., water circulators [14], sous-vide units [15–17], hot plate [18] or Incubator [19]) are bulky and require access to mains AC power, restricting their use for remote or resource limited regions. Furthermore, advanced or more integrated portable platforms [20–29] such as portable handheld reader, qcLamp, LAMP-on-a-Chip instruments often come with significantly higher costs, greater fabrication complexity and partial documentation hindering their scalability.

To overcome these challenges, we have developed an incubation device for LAMP assays that is portable, affordable (<$5), robust, easy to build and still has high temperature stability. We call this incubation device “MobileLAMP” as it can be powered using widely available 5V USB power outlets, including mobile phones. In the article, we will describe the building blocks of the MobileLAMP, its fabrication and operation, and evaluate its performance by demonstrating the detection of SARS-CoV-2 and *Salmonella enterica* serovar Typhi (*S.* Typhi). We provide a detailed head-to-head comparison with representative LAMP incubators from the literature (Table 1), demonstrating MobileLAMP’s specific advances across standardized metrics including cost, power requirements, fabrication complexity, capacity, and open-source accessibility. We share all the design files for MobileLAMP openly and believe that MobileLAMP can be widely adopted for other isothermal nucleic acid amplification assays.

## 2 Materials and methods

### 2.1 Design

**Fig 1:**
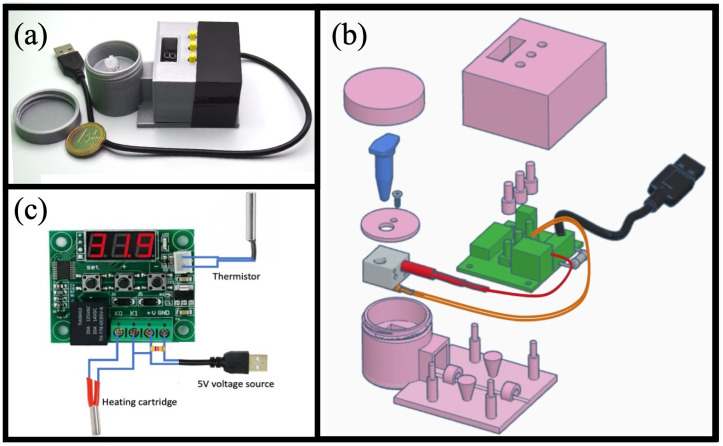
(a) Photograph of the MobileLAMP device with a one Euro coin included for size reference. (b) Exploded view of the computer-aided design (CAD) diagram illustrating the various components of the MobileLAMP. (c) Electrical schematic of the MobileLAMP, featuring the W1209 temperature controller module.

As shown in Fig 1, MobileLAMP is a simple and portable design consisting of a few components that can be grouped into three categories: i) electrical ii) mechanical iii) 3D-printed.

#### 2.1.1 Electrical components of the MobileLAMP.

**Fig 2:**
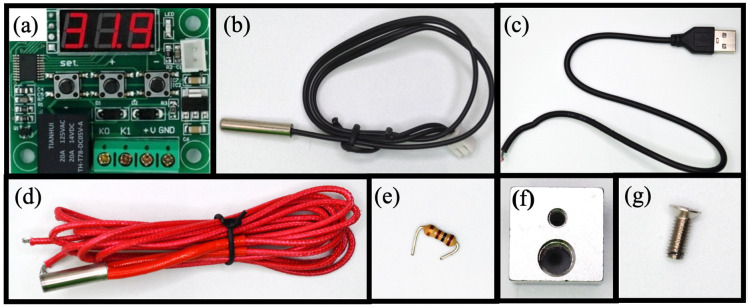
Electrical and mechanical components required for building the MobileLAMP device: (a) W1209 temperature controller module. (b) thermistor (c) USB cable (d) heater cartridge (e) 100 Ohm resistor (f) heat block (g) 3 mm screw.

The MobileLAMP device utilises a commercially available, low-cost ($1.5) thermostat module known as W1209 (Fig 2a). W1209 module is a no-code programmable thermostat that comprises three tactile switches, and a seven-segment LED display, enabling configuration of various parameters to set a desired temperature in the range of −50 °C to 110 °C (see supplementary section A for more information). As a result, the user does not need any programming knowledge to operate the MobileLAMP device. The W1209 also comes along with a temperature sensor (Fig 2b) which provides real-time temperature monitoring and feeds information to a preconfigured proportional–integral–derivative (PID) algorithm to accurately adjust the temperature.

The W1209 module operates at 5V, making it possible to power the MobileLAMP using widely available 5V USB power sources, such as mobile phones, chargers, and power banks. To facilitate this, a USB-male cable (Fig 2c) is employed as the power cable for the MobileLAMP. To provide a stable temperature for LAMP reactions, a heating cartridge (Fig 2d) commonly used in Fused Deposition Modelling (FDM) 3D printer extruders is utilised as the heat source within the MobileLAMP. A 100 ohm resistor (Fig 2e) is used in MobileLAMP to draw the minimum required current to ensure that it works with the power banks as some rechargeable power banks require a minimum current draw to maintain a steady power supply.

#### 2.1.2 Mechanical components of the MobileLAMP.

The MobileLAMP device utilises an aluminium heating block (Fig 2(f)), commonly found in FDM 3D-printer extruders, as its heating block. This block features two 6 mm diameter holes, one for holding a heating cartridge and one for a nozzle in 3D-printer extruders. These holes are repurposed in the MobileLAMP to hold the heating cartridge and a microtube containing the LAMP reaction mixture. Due to its widespread usage, the aluminium heating block can be obtained at a low-cost of less than $1. Additionally, a 3 mm screw (Fig 2(g)) is utilised in the construction of the MobileLAMP to secure the heating cartridge in the heating block as detailed in Section 3.2.

#### 2.1.3 3D-printed parts of the MobileLAMP.

**Fig 3:**
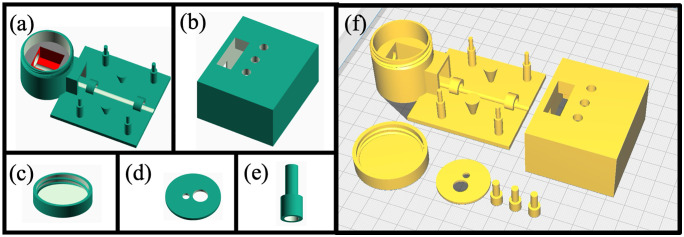
(a) to (e) Computer-aided design (CAD) diagrams of the MobileLAMP’s 3D-printed components designed using OpenSCAD software. (f) Representation of the print orientation of the MobileLAMP’s 3D-printed parts in the CURA slicing software.

**Fig 4:**
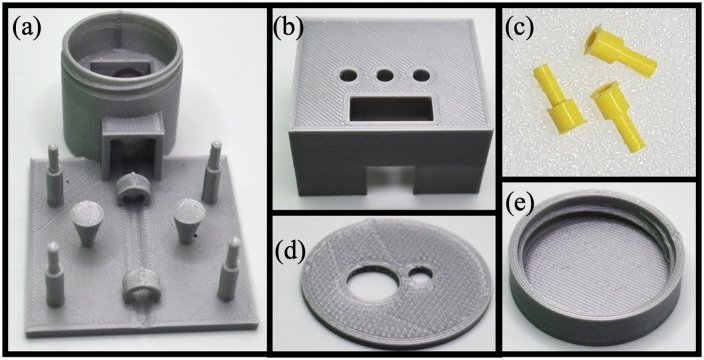
Three-dimensional (3D) printed components of the MobileLAMP using Polylactic Acid (PLA) material. **(a)** Bottom portion of the electronic enclosure. **(b)** Top portion of the electronic enclosure. **(c)** Tactile switch buttons for the W1209 module. **(d)** Circular cover for the heat block. **(e)** Heat chamber lid for the MobileLAMP.

The MobileLAMP consists of seven 3D-printed parts, designed using OpenSCAD (RRID:SCR_018870) [30], a free and open source parametric CAD software. Fig 3a to 3e show the screenshots of the CAD images of these 3D-printed parts. The bottom part of the enclosure (Fig 3a) is designed to accommodate the heat block, the temperature sensor and the W1209 thermostat electronic controller. One notable aspect of the enclosure is the jar and lid design (Fig 3c), along with a circular cover (Fig 3d) helps to minimise heat loss from the heating block to the environment. The top part of the enclosure (Fig 3b) has provision for three 3D-printed buttons (Fig 3e) which allow users to interact with the tactile switches of the W1209 and control heat block’s temperature. Additionally, the top part of the enclosure includes a window for the 7-segment LED display of the W1209 module. The CAD design files of the MobileLAMP were exported as STL files from Openscad and then converted to G-code using CURA (RRID:SCR_018898) [31], an open-source slicer program that slices 3D CAD models into 2D layers. Care was taken to print the parts without any support structures to reduce plastic waste and reduce assembly time. The G-code files were then uploaded to an Ultimaker S3 3D-printer [32] and printed using 2.85 mm diameter PLA material with the following settings: 0.2 mm layer height, 20% infill density, 0.4 mm nozzle diameter, 210°C nozzle temperature, 60°C build plate temperature, 50 mm/s print speed, and no support structures. The print orientation of the objects of the MobileLAMP are shown in Fig 3(f). Fig 4 shows these 3D-printed parts of the MobileLAMP.

#### 2.1.4 Bill of materials.

The complete Bill of Materials (BOM) for constructing the MobileLAMP is provided in Table 2, with a total component cost of $4.98.

**Table 2:** Bill of Materials to construct the MobileLAMP. Note that the cost of these 3D-printed parts is estimated assuming a $22.99 per kg spool of PLA material [33].

Component	Parameters	Number	Cost ($)	Total Cost ($)	Source Of Materials	Material Type
Electrical components						
W1209 module	5V version	1	1.5	1.5	AliExpress	NA
USB cable	Male	1	0.5	0.5	AliExpress	NA
Heating cartridge	14 ohms	1	1	1	AliExpress	Ceramic
Resistor	100 ohms	1	0.01	0.01	AliExpress	NA
Mechanical components						
Heat block	E3D V5	1	1	1	AliExpress	Aluminium
Screw	3mm	1	0.01	0.01	AliExpress	NA
3D-printed components						
Top part	N/A	1	0.275	0.275	In House	PLA
Bottom part	N/A	1	0.528	0.528	In House	PLA
Circular cover	N/A	1	0.0229	0.0229	In House	PLA
Lid	N/A	1	0.114	0.114	In House	PLA
Pins	N/A	3	0.007	0.02299	In House	PLA
Total				4.98		

### 2.2 Build instructions of the MobileLAMP

**Fig 5:**
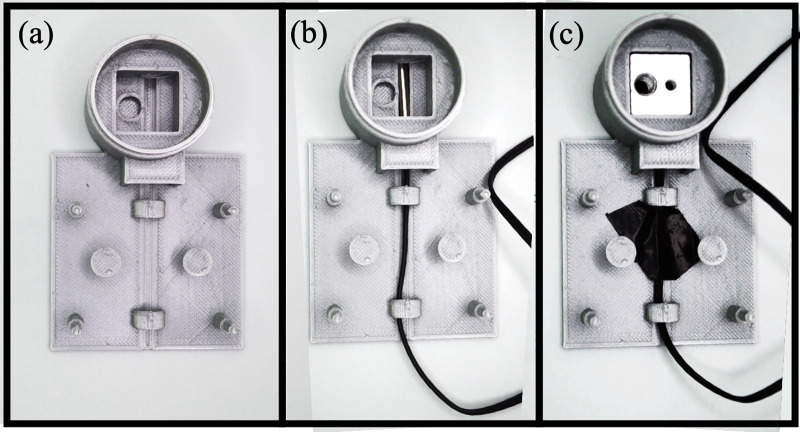
(a) 3D printed part of MobileLAMP’s bottom enclosure. (b) Thermistor is inserted into the slot. (c) Heat block is placed inside the cylindrical chamber of the bottom enclosure. Thermistor wire is held in place using black tape.

**Fig 6:**
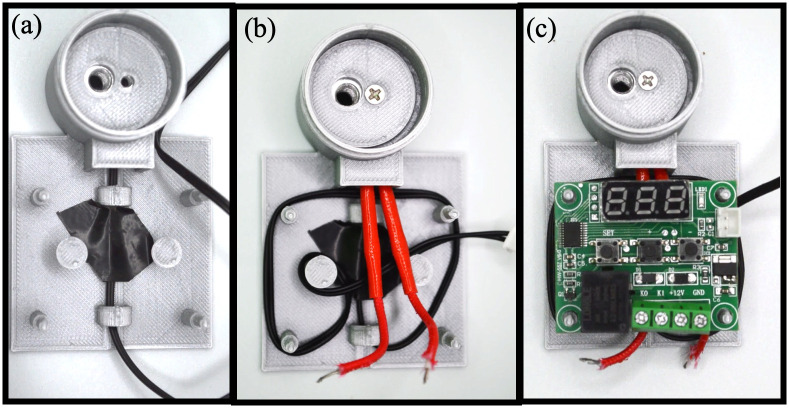
(a) A 3D-printed circular cover is fitted atop the heat block. (b) The heating cartridge is inserted into the heat block and secured in place with a 3 mm screw. (c) The W1209 module is mounted to the enclosure using the mounting pillars.

**Fig 7:**
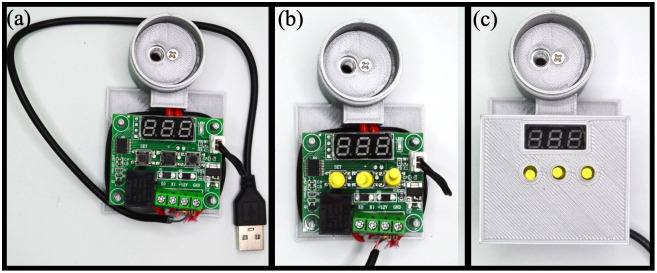
(a) Complete the electric connections according to the circuit diagram shown in Fig 1. (b) Insert the 3D-printed button pins into the tactile switches of the W1209. (c) Secure the top cover to the enclosure.

**Fig 8:**
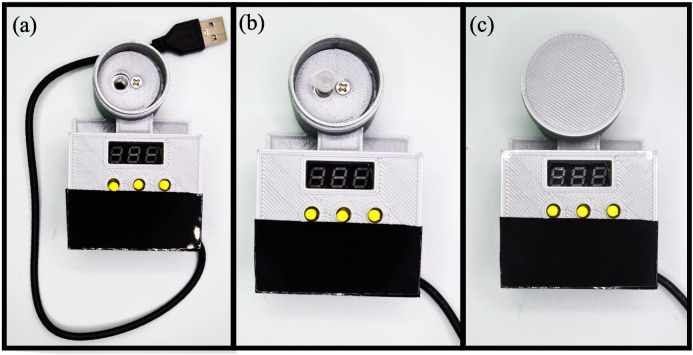
(a) The top and bottom parts of the MobileLAMP device are secured with tape. (b) The lamp reaction tube is inserted into the heat block. (c) The upper lid part is fixed to the reaction chamber of the device.

A. Begin the construction of the MobileLAMP with placing the bottom enclosure part (Fig 5a).
B. Insert the thermistor into the designated slot within the enclosure (Fig 5b).
C. Place the heat block inside the cylindrical chamber of the bottom enclosure (Fig 5c).
D. Fit the 3D-printed circular cover atop the heat block (Fig 6a).
E. Insert the heating cartridge into the heat block and secure it by tightening the 3 mm screw from the top of the circular cover (Fig 6b).
F. Mount the W1209 module to the enclosure using the four designated mounting pillars (Fig 6c).
G. Carefully follow the circuit diagram (Fig 1b) to make the necessary electrical connections (Fig 7a).
H. Insert the 3D-printed button pins into the tactile switches of the W1209 (Fig 7b).
I. Finally, fit the top part of the enclosure onto the bottom part, ensuring that the 3D-printed buttons are not obstructed (Fig 7c).
J. Secure the top and bottom parts of the enclosure together with tape to complete the construction of the MobileLAMP (Fig 8a).

### 2.3 Operational instructions of the MobileLAMP

A. To operate the MobileLAMP, connect it to a 5V USB power supply using its USB cable.
B. Set the desired temperature by adjusting the parameters P0 to P6 on the W1209 module’s settings using the 3D-printed buttons.
C. Refer to S1 and S2 Figs for detailed instructions on how to navigate the settings.
D. The W1209 module will execute the program with the selected parameters and display the real-time temperature on the seven segment LED display.
E. Wait until the MobileLAMP reaches the set temperature, as indicated on the display.
F. Once the desired temperature has been reached, place the LAMP reaction tube inside the heating block (Fig 8(b)).
G. Secure the lid by screwing it into the reaction chamber jar (Fig 8(c)).
H. Allow the LAMP reaction to proceed for the designated amount of time before removing the lid.
I. Carefully remove the LAMP reaction tube from the heating block and observe the tube for any colour changes, which will indicate the outcome of the LAMP reaction.

### 2.4 Colorimetric LAMP protocol

We performed colorimetric LAMP on synthetic targets of SARS-CoV-2 and *S.* Typhi (the bacterial pathogen that causes typhoid fever) using 2× Warmstart colorimetric LAMP (M1800, NEB, US). The RNA template of the SARS-CoV-2 N gene was synthesised using *in vitro* transcription (Hiscribe, NEB, US) and a template plasmid (Molecular Diagnostics Collection, Free Genes, Stanford University) using forward and reverse primers N-RNAF and N-RNAF (Table 3). Final working concentrations of SARS-CoV-2 primers were 1.6 µM FIP/BIP, F3/B3 0.2 µM and L3/B3 0.4 µM. *S.* Typhi synthetic template targeting *STY1607* gene [34] were synthesised (Twist Bioscience, US) and the LAMP primers (Table 3) of final concentration 3.2 µM FIP, 1.6 µM BIP, F3/B3 0.2 µM and LB/LF 0.8 µM were used [16]. 10 µl of the reaction mixture at various target concentrations along with a no DNA target blank (NTC) were incubated in MobileLAMP for 60 minutes with quick spinning every 15 minutes in a mini centrifuge to reduce any collection of condensate at the lid. These reactions were also incubated using a thermocycler (miniPCR bio™, US), for comparison.

**Table 3:** List of primers used in colorimetric LAMP for the detection of SARS-CoV-2 and *S.* Typhi.

Primer	Sequence
N-RNAF	GAAATTAATACGACTCACTATAG
N-RNAR	GACTTGATCTTTGAAATTTGGATCT
N-B3	GACTTGATCTTTGAAATTTGGATCT
N-BIP	CGCATTGGCATGGAAGTCACAATTTGATGGCACCTGTGTA
N-F3	ACCAGGAACTAATCAGACAAG
N-FIP	TTCCGAAGAACGCTGAAGCGGAACTGATTACAAACATTGGCC
N-LB	CTTCGGGAACGTGGTTGACC
N-LF	GGGGGCAAATTGTGCAATTTG
typhi_F3	AAGAGTGCGTTTGAACACTT
typhi_B3	CCCGGTCAAACTTAAGTTCC
typhi_FIP	CCTGGGGCCAAATGGCATTAACTAAGTAAGGCTGGTGAACC
typhi_BIP	AACTTGCTGCTGAAGAGTTGGAAAAAGATACCAGAGCCCGAA
typhi_LB	TCGGATGGCTTCGTTCC
typhi_LF	CAAGGGTTTCAAGACTAAGT

Note that this study used only commercially available synthetic nucleic acid targets. No human subjects, human-derived samples, or animal subjects were involved in this research.

## 3 Results

### 3.1 Temperature characterization

**Fig 9:**
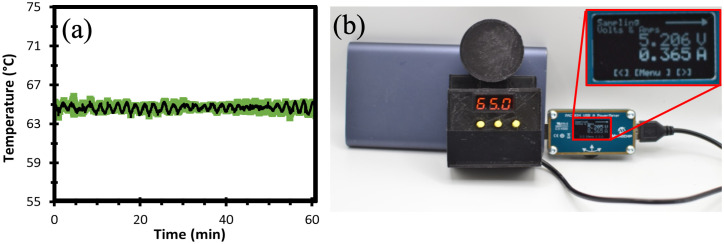
(a) MobileLAMP temperature profile at 65 °C set-point. The x-axis represents Time (min) and the y-axis represents Temperature (°C). Black line is the mean temperature value (three repeats), green shaded region denotes standard deviation from the mean. (b) The power consumption of the MobileLAMP device is measured with USB-A power meter (PAC1934 from Microchip Technology Incorporated), by connecting the power meter between the USB power source and the MobileLAMP. Current consumption of the MobileLAMP (365 mA) is for the 65°C is shown in the inset.

In this work, a temperature of 65°C is used for all LAMP reaction experiments. To achieve this temperature, the W1209 module is set to the following parameters: Target temperature set to 62.5°C, P0 set to H, P1 set to 0.5°C, P2 set to 62.5°C, P3 set to 65°C, P4 set to 62°C, P5 set to 7°C and P6 set to OFF. To validate the MobileLAMP performance, we additionally evaluated the stability of the set-point temperatures using a 10kΩ commercially available NTC thermistor (Vishay, USA) using an Arduino Uno microcontroller. The thermistor was immersed in a microtube with water and the temperature profile at 65°C was monitored for 60 minutes as shown in Fig 9(a). The mean temperature of three repeats for an hour was measured to be 64.7℃ and a standard deviation of 0.2℃ and [64.2, 65.2]°C 95% confidence interval (CI). To demonstrate the broader performance of the incubation device, we also configured the W1209 to achieve 55°C set-point with the following settings: Target temperature set to 53.5°C, P0 set to H, P1 set to 0.5°C, P2 set to 53.5°C, P3 set to 55°C, P4 set to 53°C, P5 set to 6.5°C and P6 set to OFF. The mean temperature of three repeats for an hour was measured to be 54.57°C and a standard deviation of 0.17℃ and [54.15, 54.99]°C 95% CI, as shown in S3 Fig.

### 3.2 Electrical characterization

We measured the power consumption of MobileLAMP using a USB-A power meter (PAC1934 USB-A from Microchip Technology Incorporated). The test setup involved connecting the power meter between the USB source and the MobileLAMP device, as shown in Fig 9(b). It is evident from Fig 9(b) that the MobileLAMP device draws only 365 mA of current which falls comfortably below the standard 500 mA provided by computers, mobile phones, and comparable devices for connecting USB peripherals. This configuration facilitates an operational span of up to 27 continuous hours (calculated as 10000 mAh/365 mA) for operating MobileLAMP at 65° C set-point. We found that while operating at a lower temperature of 55° C, the MobileLAMP device consumes only 305 mA per hour of current. Consequently, the same 10000 mAh battery can support 33 hours (~10000 mAh/305 mA) of operation.

### 3.3 Colorimetric LAMP against various targets

**Fig 10:**
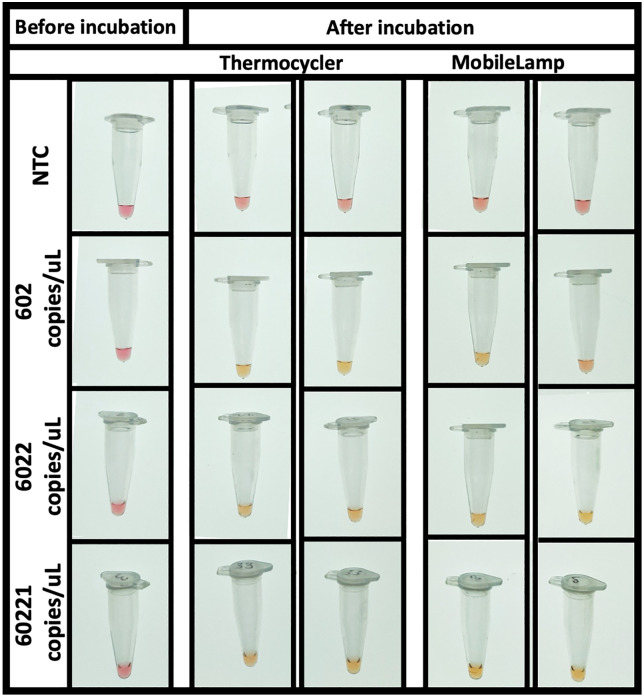
Detection of SARS-CoV-2 target using colorimetric RT-LAMP using MobileLAMP compared with thermocycler. In positive LAMP reactions, the pH indicator dye changes from pink (negative) to yellow (positive) due to acid production during DNA amplification. The detection was carried out at 602 copies/μL (second row), 6022 copies/μL (third row) and 60221 copies/μL (fourth row) compared to NTC (no template control, first row). The figure shows results before incubation (left column, all pink) and after incubation, with duplicate samples tested in both thermocycler (middle two columns) and MobileLAMP (right two columns). MobileLAMP successfully amplified the targets as indicated by colour change from pink to yellow, demonstrating equivalent performance to the commercial thermocycler. **Note:** For colour-blind accessibility, besides the distinct hue and brightness shift from dark pink to lighter yellow, positive reactions appear in the ‘After incubation’ columns for both devices at all target concentrations, while the NTC (first row) remains pink in all conditions.

**Fig 11:**
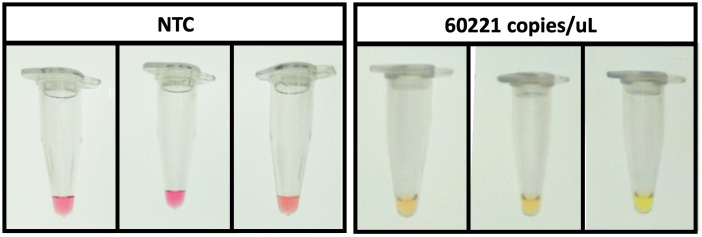
MobileLAMP for *S.* Typhi detection. NTC (no template control, left panel with three tubes) showed no colour change (remaining pink), indicating no amplification occurred. The target STY1607 gene at 60221 copies/μL (right panel with three tubes) changed colour to yellow, indicating successful DNA amplification and positive detection. **Note:** For colour-blind accessibility, besides the distinct hue and brightness shift from dark pink to lighter yellow, the positive results are shown in the right panel (three tubes), while the negative controls are shown in the left panel (three tubes).

We first performed the colorimetric RT-LAMP to detect SARS-CoV-2 RNA with the MobileLAMP and compared it with a thermocycler as shown in (Fig 10). The measurements were done in duplicate at three different target concentrations (602, 6022 and 60221 copies/µL) compared against the NTC. The reactions turned yellow from pink when the target was detected and amplified in both the devices. We next performed colorimetric LAMP assay with the MobileLAMP to successfully detect with *S.* Typhi gene *STY1607* DNA at a concentration of 60221 copies/µL as shown in Fig 11.

## 4 Discussion

### 4.1 Advantages of the MobileLAMP

The MobileLAMP device provides several advantages compared to the existing similar devices in the field of isothermal nucleic acid amplification.

#### 4.1.1 Affordability.

The bulk of the cost associated with building low-cost LAMP devices often comes from the electronic controller and heat block components. In contrast to some higher-cost incubator designs, MobileLAMP significantly reduces these costs by utilising off-the-shelf components that are both low-cost and widely available. The W1209 module used in the MobileLAMP is an affordable ($1.5) temperature controller that can be easily obtained even in single quantities. Note that the W1209 module was used in a LAMP incubation device recently [29]; however does not include detailed working of the module which we have covered in this work. The heat block used in MobileLAMP is a widely used aluminium block in FDM 3D-printers, which can be purchased for as little as $1 even in low quantities. This results in a total cost of the MobileLAMP being less than $5 including 3D-printed parts (Table 2), making it the lowest-cost instrument-based LAMP incubator available, and even more cost-effective than some instrument-free LAMP incubators.

While several incubation devices claim low cost, MobileLAMP provides a complete BOM. Most Instrument-based designs either do not provide complete BOMs (e.g., Portable Reader [20] qcLamp [21], FluoroPLUM [24]) or have significantly higher costs (FABL-8 [22]: $380, LARI: $1,500 [23], MINI: $4,900 [25], LAMP-on-a-Chip [26]: $180). The closest USB-powered alternative to MobileLAMP, qByte ($60), is 12 times more expensive. MobileLAMP’s $5 cost is achieved through strategic use of off-the-shelf components and 3D printing, eliminating the need for custom PCB fabrication or CNC machining.

#### 4.1.2 Simplicity.

The MobileLAMP is designed with a minimum number of components, making it quick and easy to build, even for non-experts. In comparison, many instrument-based LAMP devices in the literature (see Table 1) require a wide range of skills, including programming, soldering, microfluidics, CNC routing and 3D-printing, making them difficult to build. This simplicity in design makes MobileLAMP particularly well-suited for both (i) resource and budget constrained POCs where multiple units of MobileLAMP can be set up depending on the patient load (ii) integrated into home-testing kits with colorimetric LAMP.

#### 4.1.3 Scalability.

The design of the MobileLAMP is low-cost and open-source, aiding scalability of supply. While it uses technologies such as FDM 3D-printing that are not well-suited to centralised mass manufacturing, it is possible to produce in large quantities, if required, with the help of the 3D printing community through distributed manufacturing. This approach was prevalent during the COVID pandemic for PPE manufacture, and has been explored as a way to increase access to medical devices. The small size of MobileLAMP also makes it possible to print copies on low-cost 3D printers, or to print multiple units simultaneously on large 3D-printers. Its open-source design enables users to modify and customise the device to meet their specific needs, which can foster innovation and collaboration within the scientific community.

#### 4.1.4 Deployability.

MobileLAMP operates at 5V, requiring only 365 mA of current, which can be supplied by ubiquitous 5V USB powered-devices, such as mobile phones, and associated power supplies, such as battery banks, which are widely available. For example, the MobileLAMP can run on a 10000 mAh power pack for 27 hours, or the equivalent of 27 assays. This low-power requirement along with its portability makes the MobileLAMP appropriate for field use, which can increase the efficiency and speed of sample collection and analysis. It also provides the ability to perform analysis in remote locations, where laboratory and diagnostic infrastructure may be limited. For such field deployment, adherence to standard biosafety protocols, including the use of sealed reaction tubes and appropriate disposal of biological waste, is essential to prevent environmental contamination.

Recent research and reviews have highlighted the expanding role of LAMP in point-of-care diagnostics for pathogen detection in clinical and food safety applications [35,36]. MobileLAMP contributes to this diagnostic ecosystem by addressing key barriers to LAMP adoption in resource-limited settings: the combination of documented sub-$5 cost, verified 5V USB power compatibility, and complete open-source design represents a unique position among reported LAMP incubation platforms.

### 4.2 Limitations of MobileLAMP

#### 4.2.1 Limited number of tubes.

One of the limitations of the MobileLAMP device is its limited capacity. It can only hold one reaction tube at a time, which was a necessary trade-off to use the off-the-shelf heat block. While one sample is adequate for the intended application of the device, it does not allow for a negative or positive control sample to be run simultaneously. However, it is worth noting that even commercial LAMP devices [6] designed for PoC applications typically have provision for only one tube, so this limitation is not unique to the MobileLAMP device.

Despite this constraint, several practical approaches can address the control requirement. In point-of-care and laboratory settings, this limitation can be effectively managed by deploying multiple (sample, positive control and negative control) MobileLAMP devices simultaneously. This approach also provides operational redundancy and allows for parallel processing of multiple samples when needed. For applications where simultaneous controls are not critical, such as home testing or field screening, sequential processing offers a practical solution. Users can run their sample first, followed immediately by appropriate controls using the same device. We have also considered future design modifications that could accommodate multiple tubes while preserving the core advantages of simplicity and affordability. A multi-tube version could potentially hold 3–4 reaction tubes using a larger custom heat block, though this would require engineering trade-offs between cost, complexity, and performance. The cost-benefit analysis of these three options is summarised in Table 4.

**Table 4:** Cost-benefit analysis of approaches to address the control requirements.

Approach	Cost ($)	Pros	Cons
Multiple MobileLAMP units	5-15	Simultaneous processing, redundancy, scalability	Requires multiple power sources, more setup space
Multi-tube redesign	15-25 (estimated)	Single device operation, integrated controls	Increased build complexity, higher cost, custom parts
Sequential processing	5	Lowest cost, simple operation	Time delay between runs, no simultaneous controls

#### 4.2.2 Lack of heated lid.

Another limitation of the MobileLAMP device is its lack of a heated lid on top of the reaction chamber, which is commonly used in commercial LAMP devices to prevent evaporation of the reaction mixture and to maintain uniform mixing of the reagents with the sample. While the simplified design of the MobileLAMP device without the heated lid offers advantages in terms of ease of use and cost, this lack of lid can result in evaporation and non-uniform mixing of the reagents. This can be mitigated by using mineral oil on top of the reaction mixture, by using a larger volume of reaction mixture or, less conveniently, by periodically centrifuging the tube.

#### 4.2.3 Qualitative results.

One of the limitations of our MobileLAMP platform is its reliance on qualitative assessment through visual observation for determining the outcome of LAMP reactions. This contrasts with some advanced open-source LAMP platforms that incorporate real-time fluorescence detection, offering quantitative analysis capabilities [22,28]. While the MobileLAMP device effectively indicates the presence or absence of target nucleic acids through a colour change, it does not currently facilitate the quantification of nucleic acid concentrations within samples. For many point-of-care and home testing scenarios such as food safety screening, rapid field screening during disease outbreaks, environmental monitoring, and educational demonstrations, simple yes/no answers are sufficient. However, certain clinical and research applications require quantitative information such as viral load monitoring and gene expression analysis.

Despite these limitations, quantitative capabilities can be integrated into future iterations of MobileLAMP while maintaining core advantages of simplicity and affordability. The most promising near-term approach involves smartphone-based colorimetric analysis, which could provide semi-quantitative results without requiring additional hardware cost beyond a standard smartphone. Alternative upgrade paths include simple photodiode-LED systems for basic photometric measurements and fluorescence detection for research-grade quantification, which we estimate would add approximately $10 to $15 to the total component cost.

Furthermore, while our laboratory validation demonstrates the engineering robustness of MobileLAMP, comprehensive field trials in resource-limited settings with non-specialist operators and clinical samples remain an important objective for future work to fully assess its practical utility and durability under varied environmental stressors.

## Conclusions

MobileLAMP provides the highest level of functionality that we have observed for a LAMP device with an equivalent part cost of <$5. It has the additional advantage of simple assembly, achieved through repurposing off-the-shelf components and leveraging 3D printing to produce a device that has favourable technical characteristics for isothermal amplification at point of care. MobileLAMP can hold an average temperature of 64.7℃ with a standard deviation of 0.2℃ over the course of an hour and can run on a 10000mAh power pack for 27 hours, or the equivalent of 27 assays. The trade-offs include being limited to a single tube capacity and lack of heated lid. However, neither of these preclude its use to successfully run LAMP reactions for field research and educational use, and it is amenable to distributed mass manufacture in a crisis situation, contingent on the continuation of supply chains for consumer electronics. The designs have been made available under an Open Hardware License to encourage replication and improvement by other researchers.

## Supporting information

S1 Fig To set a target temperature on the MobileLAMP device, follow these steps: (a) Press the left button once to enter into the temperature change mode. (b) Use the middle button to increase the temperature. (c) Use the right button to decrease the temperature.
(TIFF)

S2 Fig (a) To access the additional settings of the MobileLAMP device, press and hold the left button. (b) Use the middle and right buttons to navigate through the settings menu. (c) Once the desired setting is identified, press the left button to enter it, and then use the middle and right buttons to adjust the value of the selected setting. Press the left button to exit the menu. See S1 Table for setting these values.
(TIFF)

S3 Fig MobileLAMP temperature profile at 55 °C set-point. The x-axis represents Time (min) and the y-axis represents Temperature (°C). Black line is the mean temperature value (three repeats), green shaded region denotes standard deviation from the mean.
(TIFF)

S1 Table Various settings of the W1209 module with the settings ranges and default values. (PDF)

S1 Data Raw data used in plotting Fig. 9a. (XLSX)

S2 Data Raw data used in plotting S3 Fig. This project contains the following extended data: – MobileLAMP_bottom_part.scad – MobileLAMP_bottom_part.stl – MobileLAMP_circular_cover.scad- MobileLAMP_circular_cover.stl – MobileLAMP_lid.scad. - MobileLAMP_lid.stl – MobileLAMP_pins.scad – MobileLAMP_pins.stl – MobileLAMP_top_part.scad – MobileLAMP_top_part.stl The above CAD and STL files are also available at: https://github.com/MohiniBhupathi/MobileLAMP.
(XLSX)
