# Supplementary figures and images for: MobileLAMP: A low-cost, portable incubation device for isothermal nucleic acid amplification
Source: PLoS One. 2026 Apr 16;21(4):e0346874. doi: 10.1371/journal.pone.0346874 (PMC13086327; doi:10.1371/journal.pone.0346874)

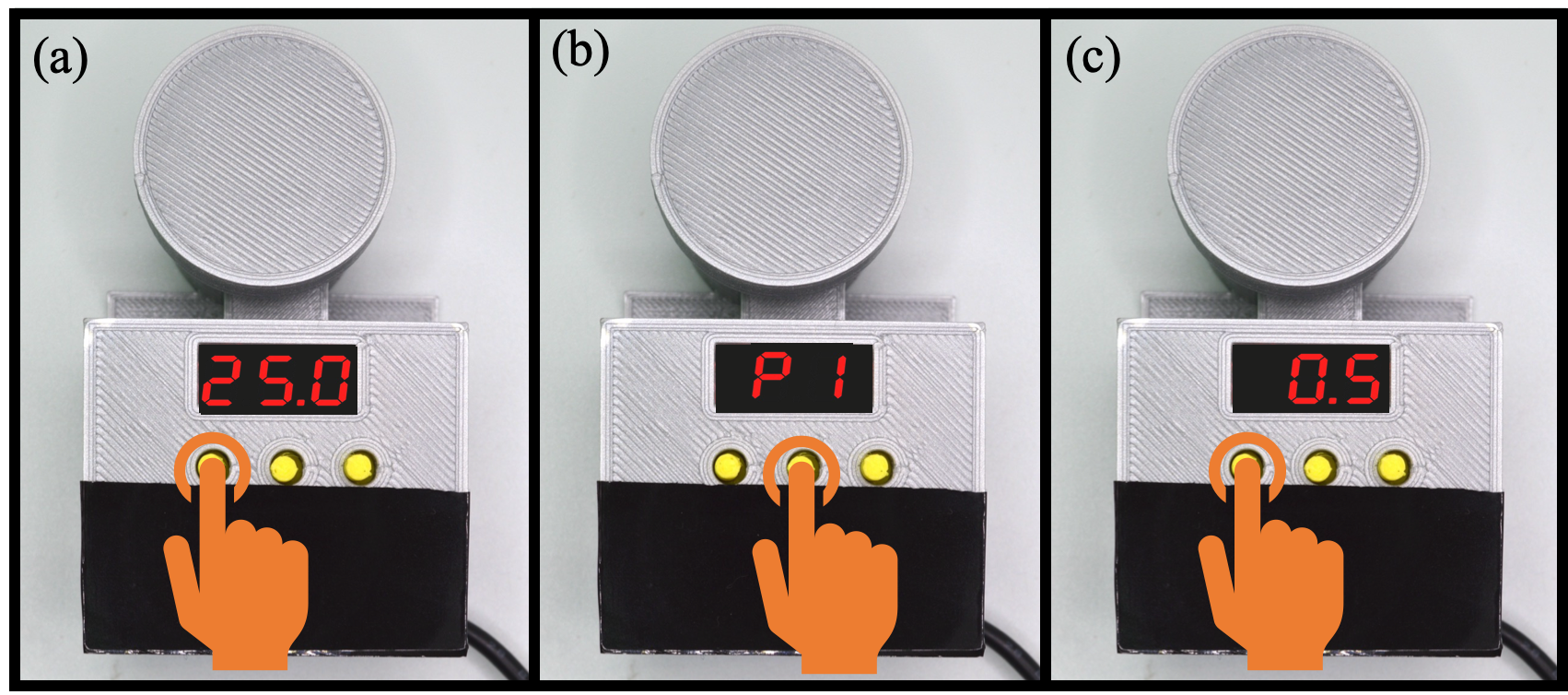

Supplement: S1 Fig — (b) Use the middle button to increase the temperature. (c) Use the right button to decrease the temperature. (TIFF) [file pone.0346874.s001.tiff]

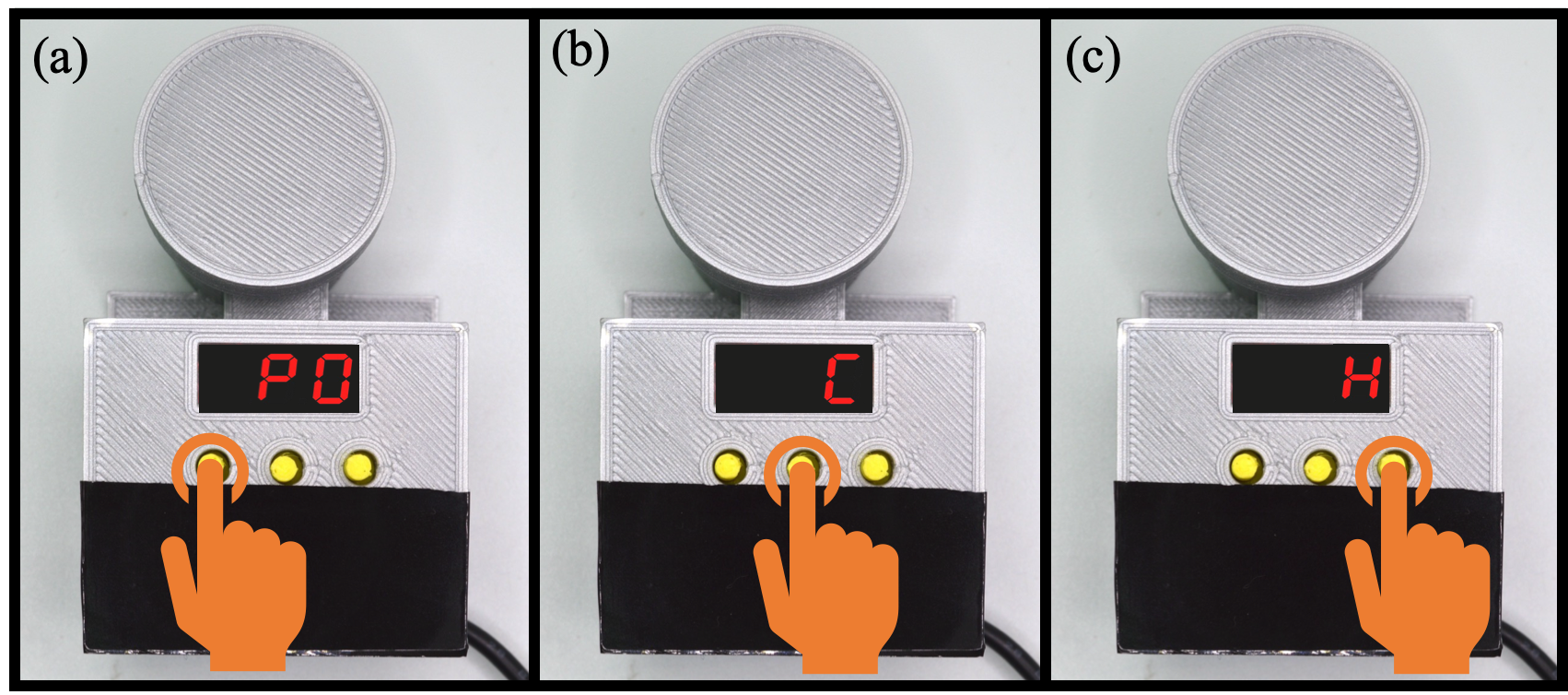

Supplement: S2 Fig — (b) Use the middle and right buttons to navigate through the settings menu. (c) Once the desired setting is identified, press the left button to enter it, and then use the middle and right buttons to adjust the value of the selected setting. Press the left button to exit the menu. See S1 Table for setting these values. (TIFF) [file pone.0346874.s002.tiff]

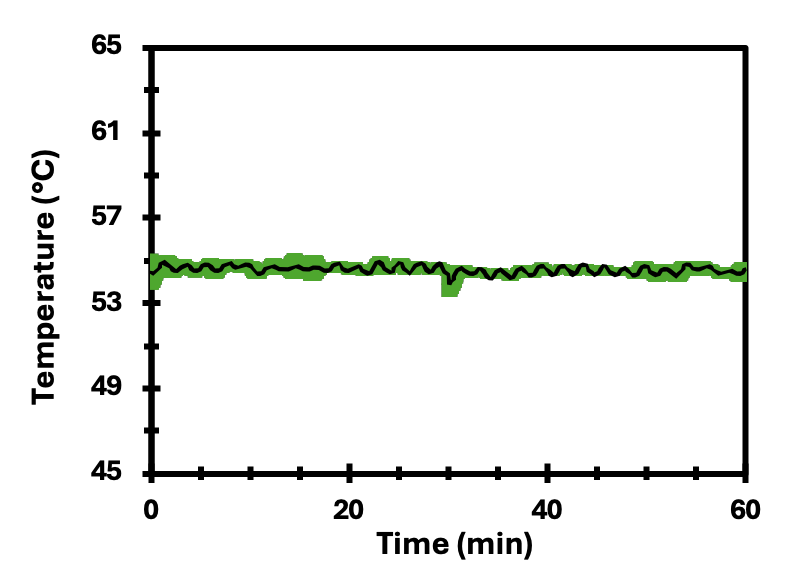

Supplement: S3 Fig — The x-axis represents Time (min) and the y-axis represents Temperature (°C). Black line is the mean temperature value (three repeats), green shaded region denotes standard deviation from the mean. (TIFF) [file pone.0346874.s003.tiff]
