# Supplementary material for: MobileLAMP: A low-cost, portable incubation device for isothermal nucleic acid amplification
Source: PLoS One. 2026 Apr 16;21(4):e0346874. doi: 10.1371/journal.pone.0346874 (PMC13086327; doi:10.1371/journal.pone.0346874)
Supplement: S1 Table — (PDF) [file pone.0346874.s004.pdf]

Code	Description	Setting Range	Default
P1	Heating/Cooling Mode	H/C	C
P2	Hysteresis (Return difference) Degrees	0.1°Cto 40°C	2
P3	Set Upper Temperature Limit (Degrees)	-50C to 110°C	110
P4	Set Lower Temperature Limit (Degrees)	-50°Cto 110°C	-50
P5	Temperature Offset Correction (Calibration) Degrees	-15°Cto +15°C	0
P6	Factory Reset (Can also press + and – for 5 seconds)	C/H	C
